# Indications for chemoradiotherapy in older patients with locally advanced head and neck cancer in Japan: a questionnaire survey in the JCOG head and neck cancer study group

**DOI:** 10.3389/fonc.2024.1441056

**Published:** 2025-01-08

**Authors:** Koichi Yasuda, Naomi Kiyota, Kazuto Matsuura, Satoshi Saito, Yoshitaka Honma, Yoshinori Imamura, Kaoru Tanaka, Sadamoto Zenda, Takuma Onoe, Takeshi Kodaira, Satoshi Kobayashi, Hidefumi Aoyama, Nobuhiro Hanai, Akihiro Homma

**Affiliations:** ^1^ Department of Radiation Oncology, Hokkaido University Hospital, Sapporo, Japan; ^2^ Department of Medical Oncology and Hematology, Cancer Center, Kobe University Hospital, Kobe, Japan; ^3^ Department of Head and Neck Surgery, National Cancer Center Hospital East, Kashiwa, Japan; ^4^ Department of Radiation Oncology, Saitama Medical University International Medical Center, Hidaka, Japan; ^5^ Department of Head and Neck, Esophageal Medical Oncology, National Cancer Center Hospital, Tokyo, Japan; ^6^ Department of Medical Oncology and Hematology, Kobe University Hospital, Kobe, Japan; ^7^ Department of Medical Oncology, Kindai University Faculty of Medicine, Osaka-Sayama, Japan; ^8^ Department of Radiation Oncology and Particle Therapy, National Cancer Center Hospital East, Kashiwa, Japan; ^9^ Division of Medical Oncology, Hyogo Cancer Center, Akashi, Japan; ^10^ Department of Radiation Oncology, Aichi Cancer Center Hospital, Aichi, Japan; ^11^ Department of Gastroenterology, Kanagawa Cancer Center, Yokohama, Japan; ^12^ Department of Radiation Oncology, Faculty of Medicine and Graduate School of Medicine, Hokkaido University, Sapporo, Japan; ^13^ Department of Head and Neck Surgery, Aichi Cancer Center Hospital, Nagoya, Japan; ^14^ Department of Otolaryngology-Head & Neck Surgery, Faculty of Medicine and Graduate School of Medicine, Hokkaido University, Sapporo, Japan

**Keywords:** older patients, head and neck cancer, chemoradiotherapy, radiotherapy, cisplatin

## Abstract

**Introduction:**

Chemoradiation therapy (CRT) with concurrent high-dose cisplatin (CDDP) is one of the standard treatment options for locally advanced head and neck cancer. Since the indications specific to the older population have not been reported, we conducted a multicenter survey on the indications.

**Methods:**

In April and May 2023, a questionnaire survey was emailed to all institutions belonging to the JCOG-HNCSG, consisting of 37 institutions.

**Results:**

The major factors influencing the indications for high-dose CDDP were renal function and performance status (PS). The majority agreed that the treatment is administered to patients aged 65–74 years with PS 0–1 and 65–74 years with eGFR ≥60 (ml/ min/1.73m2), and not in patients aged ≥75 years with PS 2, ≥80 years with PS 1, and ≥65 years with eGFR <60. Regarding weekly CDDP, the majority agreed that the treatment is not conducted in patients aged ≥75 years with PS 2, ≥65 years with eGFR <40, and ≥70 years with eGFR <50.

**Discussion:**

In Japan, where CRT is actively performed even among older people, a survey was conducted to determine its indications. Renal function and PS were considered important, and comorbidities, such as heart failure, were considered while determining the indication. These results will help define the eligibility criteria for prospective studies on CRT in older patients.

## Introduction

Chemoradiation therapy (CRT) with concurrent high-dose cisplatin (CDDP) is one of the standard treatment options for locally advanced head and neck cancer (1–3). Compared with younger patients, older patients have a poorer general condition and more varied comorbidities (4–9). They are often unable to use high-dose CDDP, in which case CRT with other less-toxic chemotherapies or radiotherapy (RT) alone is employed (10). Some literature reviews and expert panel opinions from specific countries have reported on the indications for CRT with high-dose CDDP (11–13). However, these findings are not specific to the older population. Japan has the world’s oldest population (14), with a life expectancy of 84.3 years (15). More than half of head and neck cancer patients in Japan are older than 65 years of age (16), which is defined as elderly in many countries. Little is known about which older patients are eligible for high-dose CDDP or less-toxic chemotherapy, which has become an important and globally common clinical question in recent years.

We conducted a questionnaire survey among institutions belonging to the Japan Clinical Oncology Group Head and Neck Cancer Study Group (JCOG-HNCSG) regarding indications for CRT in older people. These results provide important insights into the indications for CRT for head and neck cancer in older individuals in Japan and other countries with aging populations.

## Methods

In April and May 2023, a questionnaire survey was emailed to all institutions belonging to the JCOG-HNCSG, consisting of 37 institutions. We asked each institution to complete a questionnaire with three physicians: one surgeon, one radiation oncologist, and one medical oncologist. Thus, we expected a maximum of 111 responses (3 physicians from each of the 37 facilities). The older patients targeted for the survey had the following characteristics:

- Age ≥65 years.
- Primary sites in the oropharynx, hypopharynx, or larynx.
- Squamous cell carcinoma.
- Stage III to IVB for p16-negative oropharyngeal, hypopharyngeal, and laryngeal cancers, and stage III for p16-positive oropharyngeal cancer (UICC-TNM, 8^th^ edition).
- Undergone definitive radiation therapy or chemoradiotherapy (excluding postoperative or palliative radiation therapy).

Treatment options assumed in the survey were as follows:

- High-dose cisplatin (CDDP 100 mg/m^2^, once every 3 weeks, 3 courses) + RT (intensity-modulated radiation therapy [IMRT], 70 Gy/35 fractions).
- Less-toxic chemotherapy + RT.
- Weekly CDDP (40 mg/m^2^) + RT (IMRT, 70 Gy/35 fractions).
- Cetuximab + RT (IMRT, 70 Gy/35 fractions).
- Carboplatin (CBDCA) + RT (IMRT, 70 Gy/35 fractions).
- Docetaxel + RT (IMRT, 70 Gy/35 fractions).
- RT alone (IMRT, 70 Gy/35 in fractions).

The questionnaire was conducted in Japanese. Its English translation is provided in the **Supplementary Materials**. Considering the exclusion of 1 standard deviation (SD) on one side, the majority was defined as an agreement of ≥85%.

For each question, differences by physician’s department were analyzed using the chi-square test with P < 0.05 considered statistically significant. JMP Pro v17.0.0 (SAS Institute Inc., Cary, NC, USA) was used for the statistical analysis.

## Results

**Figure 1:**
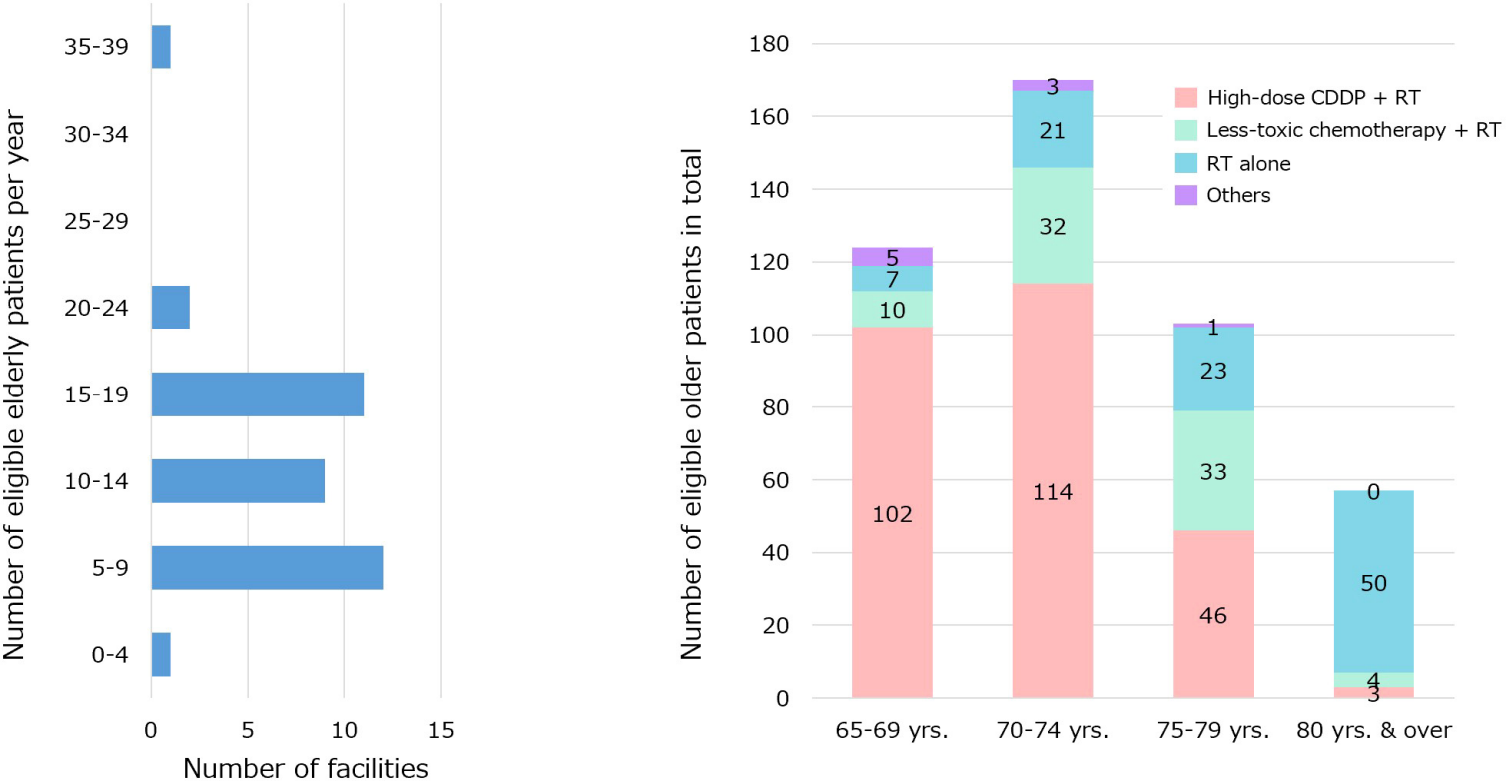
The annual number of eligible older patients at each institution and the number of patients in the entire study group were categorized according to age and treatment.

Responses were obtained from 36 facilities and 84 physicians. The response rates were 97% for the number of facilities and 76% for the number of physicians. The number of surgeons, radiation oncologists, and medical oncologists was 41, 24, and 19, respectively. The annual number of eligible older patients at each institution and the number of patients in the entire study group (categorized according to age and treatment) are shown in **Figure 1**.

**Figure 2:**
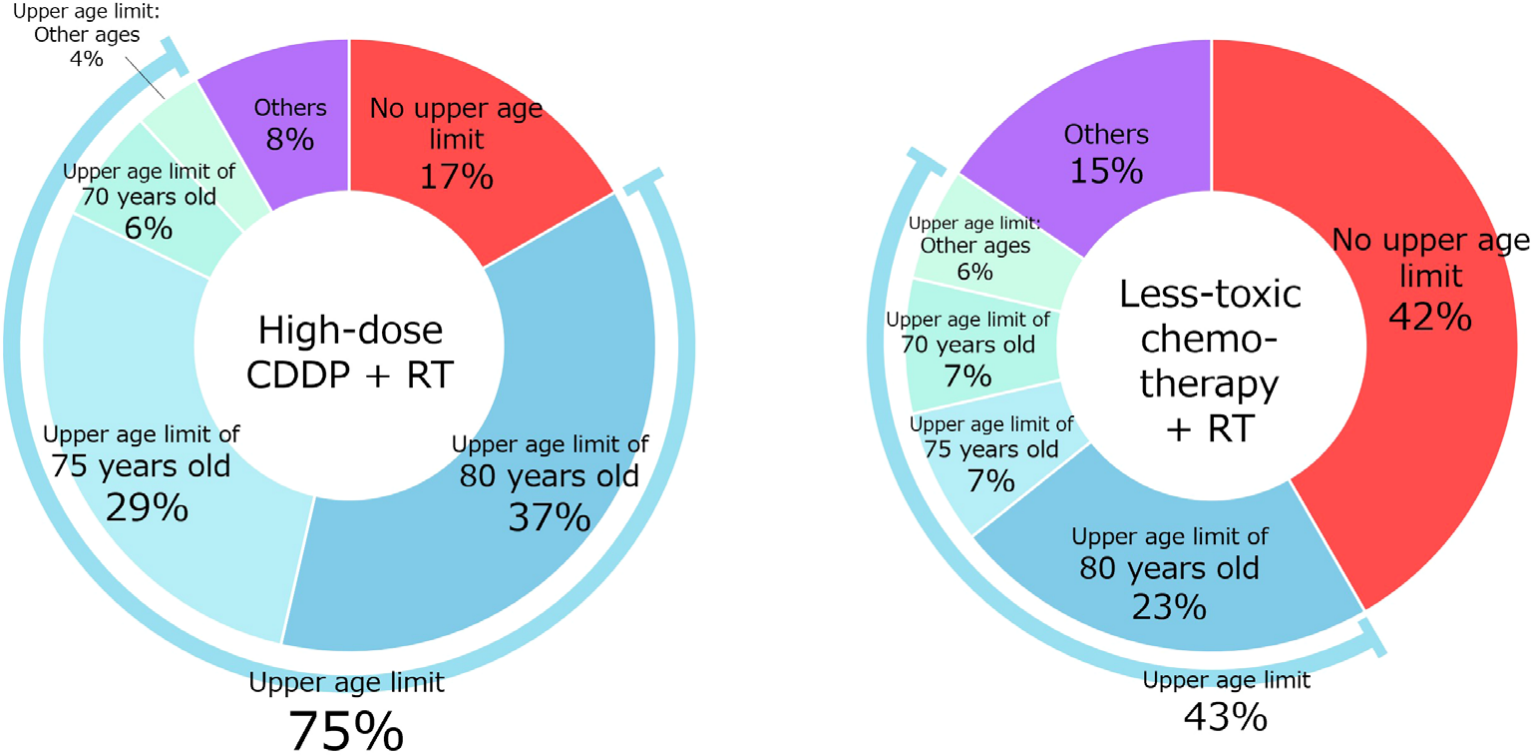
Setting an upper age limit for CRT with high-dose CDDP or less-toxic chemotherapy.

Q1 concerned the setting of an upper age limit (**Figure 2**). Regarding high-dose CDDP, 14 physicians (17% of physicians agreed) did not set an upper age limit, whereas 31 (37%) and 24 (29%) set upper age limits of 80 and 75 years, respectively. Regarding less-toxic chemotherapies other than high-dose CDDP, 35 (42%) physicians did not set an upper age limit, and 19 (23%) and 6 (7%) physicians set upper age limits of 80 and 75 years, respectively.

**Figure 3:**
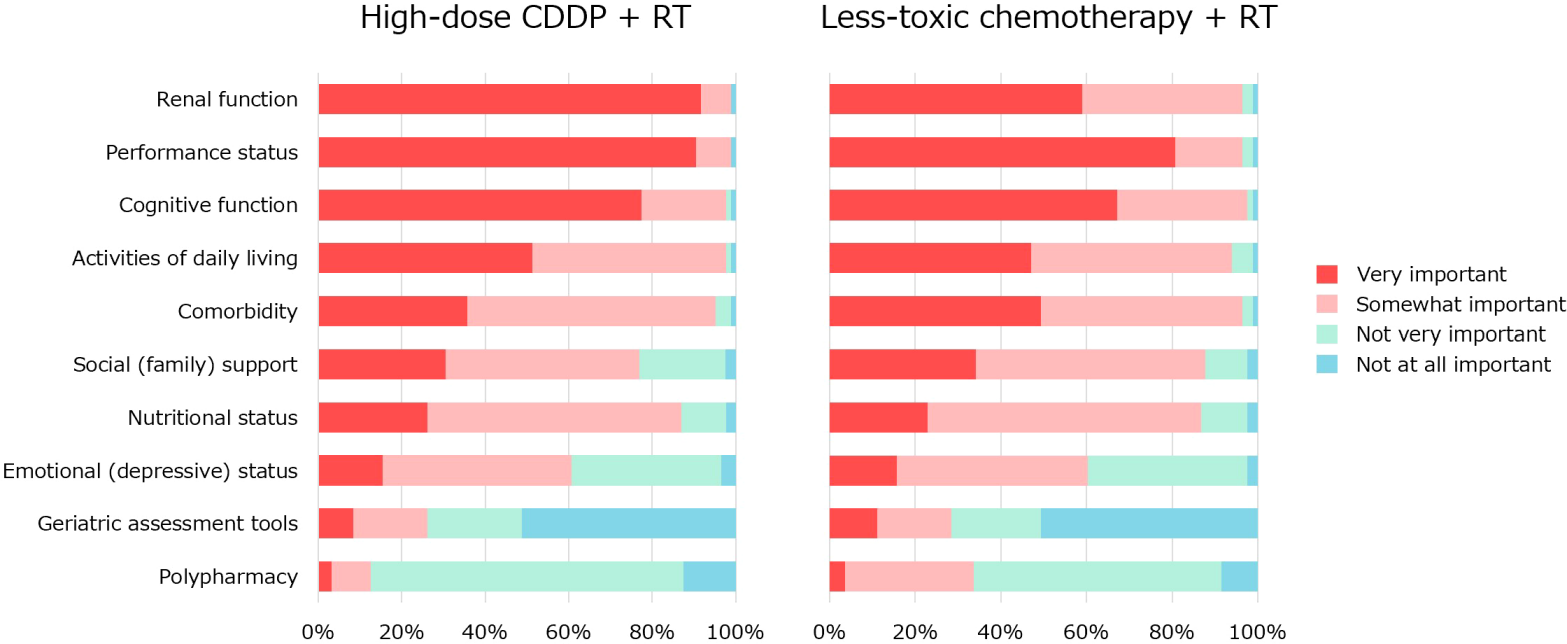
The factors influencing the indication for CRT with high-dose CDDP or less-toxic chemotherapy.

Q2 concerned factors influencing the indications (**Figure 3**). The questionnaire items were selected from previous reports (4–8). Regarding high-dose CDDP therapy, renal function (92%) and performance status (PS) (90%) were considered very important factors by the majority. Renal function (99%), PS (99%), cognitive function (98%), activities of daily living (98%), comorbidities (95%), nutritional status (87%) were considered somewhat important. Regarding less-toxic chemotherapy for older patients who were not eligible for high-dose CDDP, no significant factors were agreed upon by more than 85% of the physicians. PS (95%), cognitive function (95%), renal function (95%), comorbidities (95%), activities of daily living (93%), social (family) support (86%), and nutritional status (86%) were considered somewhat important.

Q3 concerned the type of less-toxic chemotherapy to be used in older patients who were not eligible for high-dose CDDP. (**Supplementary Figure S1**). Weekly CDDP (18%), carboplatin (17%), cetuximab (4%), and docetaxel (1%) were more frequently administered, in that order.

**Figure 4:**
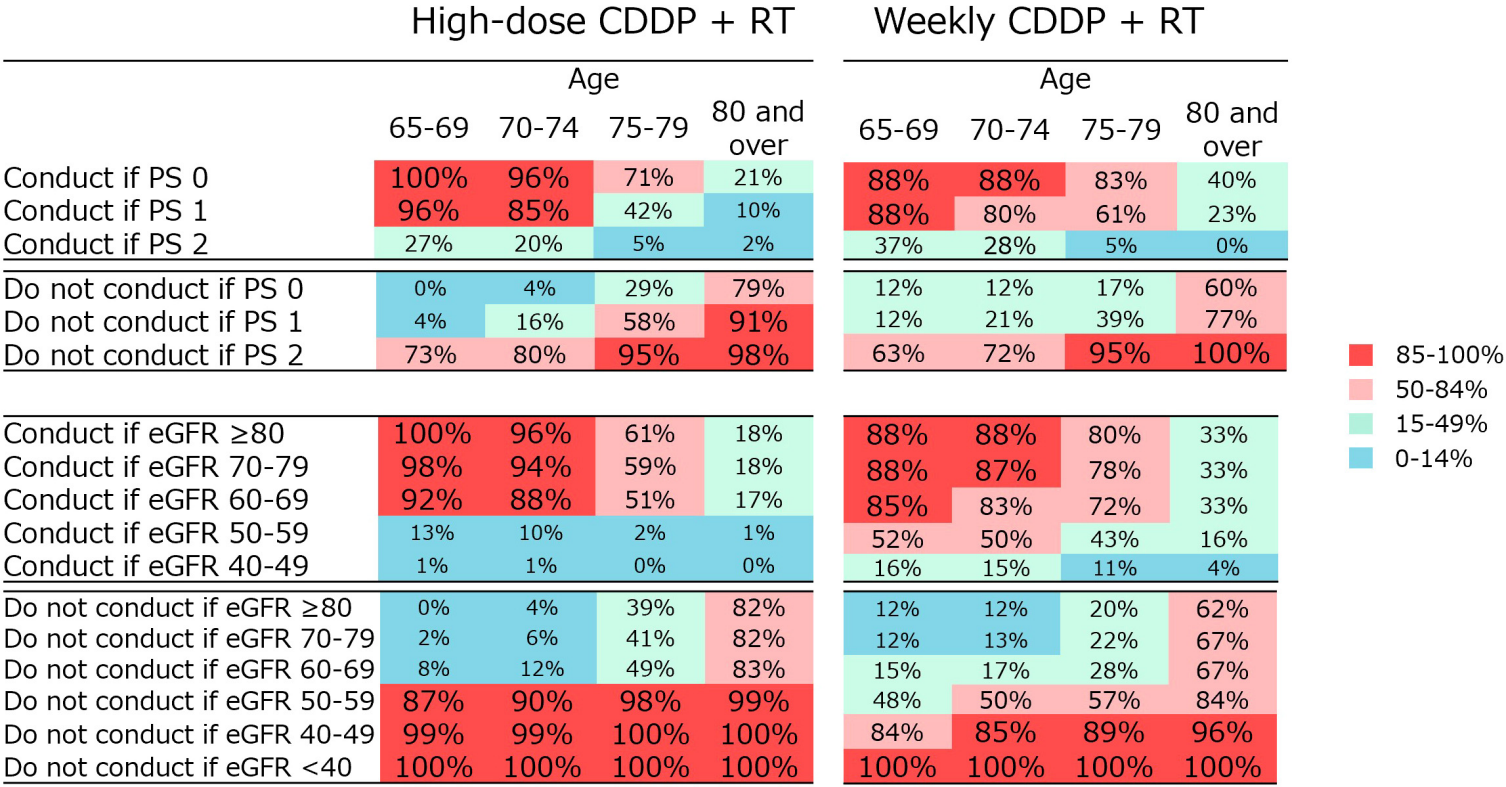
Percentage of physician agreement on whether high-dose CDDP + RT or weekly CDDP + RT should be administered depending on various PS or renal function.

The physicians were asked how the decision on the indication for CRT varied according to PS in Q4 and renal function in Q5. A summary of these results is presented in **Figure 4**. Regarding high-dose CDDP, the majority agreed that the treatment is conducted in patients aged 65–74 years with PS 0–1 (85–100%) and patients aged 65–74 years with eGFR ≥60 (ml/min/1.73m^2^) (88–100%), and not in patients aged ≥75 years with PS 2 (95–98%), ≥80 years with PS 1 (91%) and ≥65 years with eGFR <60 (87–100%). Regarding weekly CDDP therapy for the older people who are not eligible for high-dose CDDP, the majority agreed that the treatment is not conducted in patients aged ≥75 years with PS 2 (95–100%), ≥65 years with eGFR <40 (100%), and ≥70 years with eGFR <50 (85–96%).

**Figure 5:**
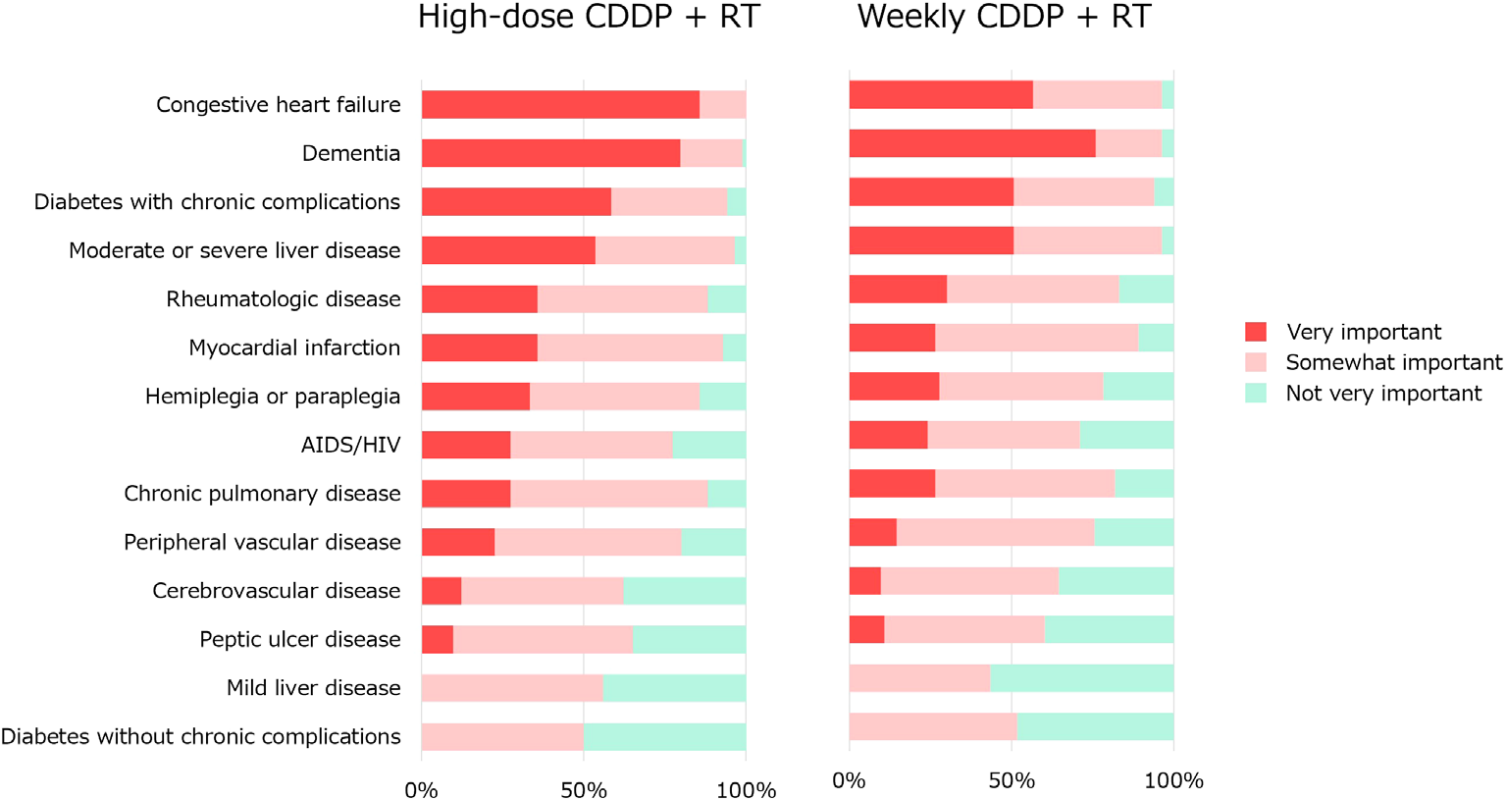
Impact of comorbidities on the indication for CRT with high-dose or weekly CDDP.

Q6 concerned the impact of comorbidities (**Figure 5**). These comorbidity items were based on Charlson Comorbidity Index (CCI) (17). Regarding high-dose CDDP, congestive heart failure is considered an important factor in the majority. Congestive heart failure (100%), dementia (99%), moderate to severe liver disease (96%), diabetes with chronic complications (94%), myocardial infarction (93%), chronic pulmonary disease (88%) and hemiplegia or paraplegia (86%) were considered somewhat important. Regarding less-toxic chemotherapy for older patients who were not eligible for high-dose CDDP therapy, no significant factors were agreed upon by more than 85% of the physicians. Congestive heart failure (96%), dementia (96%), moderate-to-severe liver disease (96%), diabetes with chronic complications (94%), and myocardial infarction (89%) were considered somewhat important.

The survey comprised 69 questions. Of these, four questions showed statistically significant differences among physicians by department (**Supplementary Figure S2**); for example, the questions concerning PS and renal function for older people aged 75–79 years when high-dose CDDP was administered.

## Discussion

CRT with high-dose CDDP is one the standard treatment options for locally advanced head and neck cancer. However, meta-analyses have not shown additional benefits of chemotherapy over RT alone in patients aged >70 years (1), and there are known variations among countries and facilities regarding the use of CRT or RT in older people (10). Several expert opinions generally concluded that high-dose CDDP therapy is relatively contraindicated in patients aged > 70 years (11–13). However, the institutions included in this survey actively used high-dose CDDP therapy, even in patients aged > 70 years. This trend was consistent with the results of a domestic database study (18). In Japan, which has the world’s oldest population (14), the number of healthy and active older people is increasing in contrast with the traditional image of the older people as inactive (19). Research conducted in older people recommends that various factors other than chronological age should be considered while making treatment decisions for patients (4–9), and it is assumed that this approach is widely accepted in Japan.

Here, 75% of physicians administering high-dose CDDP and 43% administering less-toxic chemotherapy reported having an upper age limit, suggesting that age is of some importance. The most common upper age limit was 80 years, followed by 75 years. Previous expert opinions set the age of 70 years as a relative contraindication (11–13), but in clinical practice in Japan, that age may be set a little higher. The results suggest that less-toxic chemotherapy is more tolerable in the older population.

Administrations of weekly CDDP, followed by those of CBDCA, cetuximab, and docetaxel, was the most frequently used drug in less-toxic chemotherapy. Interestingly, there was greater agreement with weekly CDDP therapy for patients who were not eligible for high-dose CDDP therapy. JCOG1008 trial showed that CRT with weekly CDDP was not inferior and less toxic than CRT with high-dose CDDP, although the study was conducted in postoperative patients with head and neck cancer (20). In the ConCERT trial, the non-inferiority of CRT with weekly CDDP in definitive chemoradiation for locally advanced head and neck cancer was recently reported (21). There are some reports on CRT with weekly CDDP for older people or patient cohorts including older people (22, 23). The low toxicity of weekly CDDP may make it the preferred treatment for the older population.

Differences in the indications for CRT according to age, renal function, and PS were surveyed in detail. The older the patient and the higher the PS, the less likely the treatment is to be indicated. For renal function, eGFR values of 60 and 50 seem to be the threshold values. These factors are complex and may make it challenging to determine a simple cutoff value in older people.

In previous reports on indications for CRT with high-dose CDDP (11–13), there was no mention of geriatric assessment (GA) tools. However, the importance of GA tools has been repeatedly emphasized in the treatment of head and neck cancer in older people (4–8). Therefore, the GA tool was included as an item in our survey of factors influencing the indications for CRT (Q2). Our results showed that GA was the second least important tool (**Figure 2**). Because we did not ask why the GA tool is less important, we do not know the exact reason for this. The international survey reported by Dale et al. is informative. In their survey, 29% reported the use of specific validated GA tools, while 69% reported an informal assessment based on their judgment. Barriers to implementing GA were identified as lack of support staff, time, knowledge, and clarity about which GA tools to use (24). The majority of physicians who responded to our survey may not have used GA tools in their daily clinical practice, which may explain why they gave such low ratings. A systematic review by Hamaker et al. found that GA resulted in 31% of patients changing their treatment course (25). Another systematic review by Anwar et al. showed that GA-based management significantly reduces toxicity (26). Further clinical research with older people, especially in Japan, should be encouraged to promote GA and GA-based management and establish its clinical significance.

Only 1% of respondents reported that they frequently used docetaxel. An international survey conducted in 2018 showed that for less-toxic chemotherapy other than high-dose CDDP and cetuximab in definitive CRT in older patients, weekly CDDP, carboplatin, and carboplatin plus 5-fluorouracil were used in that order. The others were mitomycin, paclitaxel, and capecitabine, but not docetaxel (10). This result is similar to that of our survey. The results of the DHANUSH trial by Patel et al. on CRT with docetaxel for cisplatin-unsuitable patients with head and neck cancer were published in January 2023 (27). A single-center randomized phase II/III trial compared RT alone versus CRT with docetaxel. The superiority of OS of the latter was confirmed during the study, and the trial was stopped. This survey was conducted from April to May 2023, just after publication, so it was possible that the study results were not fully incorporated into the treatment decision process of the physicians in our survey. Not only was the percentage of patients using docetaxel not high, but the percentage considering future use of docetaxel was not high. The DHANUSH trial differed significantly from the participants of this survey because it included approximately 40% of postoperative patients, more than one-third of patients with oral cancer (CRT is rarely performed in Japan), and a small proportion of older patients (approximately 16%). In addition, severe mucositis was more than doubled with RT alone, which may raise concerns about its use in older patients; it was not a multicenter study, and long-term follow-up data is lacking, which may have led to a cautious view of its future use. This study has several limitations. First, only 19 medical oncologists responded to the survey, accounting for only about a quarter of the total number of respondents; this limited the applicability of our results. Ideally, the results of this study involving cisplatin administration would be truly meaningful if all responses were received only from medical oncologists. However, the survey was conducted among physicians who practiced CRT, and it did not actually include many medical oncologists. This may be representative of the real-world situation in Japan, where cancer treatment has been conventionally performed by departments specializing in specific organs (such as otorhinolaryngology). The department of medical oncology was primarily responsible for cytotoxic chemotherapy in 13% cases of head and neck cancer, whereas the department of otorhinolaryngology managed 76% cases, even though the number of medical oncologists providing multidisciplinary cancer treatment in the field of head and neck oncology has been increasing (28). As shown in **Supplementary Figure S2**, compared with surgeons and radiation oncologists, medical oncologists were less likely to recommend high-dose cisplatin for elderly patients under certain conditions. Note, however, that this limitation is mitigated by the non-significant differences between the physician departments for 65 (94%) of the 69 questions. Second, the survey was conducted only among the institutions that participated in JCOG-HNCSG in Japan. In Japan, the “Head and Neck Cancer Registry Japan” conducted by the Japan Society for Head and Neck Cancer is the largest real-world database, with 215 facilities participating as per the 2020 report (16). The 36 facilities included in this survey represent only 17% of the total number of facilities in the national registry. It is assumed that many medium-to-large institutions are included in the JCOG-HNCSG and that the situation in small facilities is not well-reflected in the survey. Third, this was a survey of decisions (opinions) and not of the actual number of patients treated. The actual patient’s PS, renal function, comorbidities, and the treatments that were administered were not investigated; it is only an expert opinion. Fourth, the total number of patients with head and neck cancer per year was not surveyed, so the fraction of the population covered by this study’s participants was missing. Yasuda et al. analyzed the Head and Neck Cancer Registry of Japan, in which most JCOG-HNCSG facilities participated (18). From 2011 to 2014, the total number of patients with oropharyngeal, hypopharyngeal, and laryngeal cancers was 13,567. There were 7,002 cases of locally advanced cancer, and 2,912 of them underwent definitive RT or CRT. Of these patients, 1,057 were aged 70 years or older, which corresponds to 8% of the total 13,567 patients. The present study, unlike the one conducted in 2023, targeted patients aged 65 years and older, so a precise estimate could not be made. Still, it was roughly estimated that approximately 10% of the annual number of patients with oropharyngeal, hypopharyngeal, and laryngeal cancers in Japan may be covered in this study.

This report is valuable for a detailed investigation of the factors related to the decision regarding CRT indications for older people. With the increasing number of older people worldwide (15), there may be more prospective clinical trials in older people. These results provide important information for defining the eligibility criteria for prospective studies on CRT in older people.

## Conclusion

In Japan, where CRT is actively performed even among older people, a survey was conducted to determine its indications. Renal function and PS were considered important, and comorbidities, such as heart failure, were considered while determining the indication. These results provide important information for defining the eligibility criteria for prospective studies on CRT in older people.

## Data Availability

The original contributions presented in the study are included in the article/**Supplementary Material**. Further inquiries can be directed to the corresponding author.

## References

[1] Lacas B Carmel A Landais C Wong SJ Licitra L Tobias JS. Meta-analysis of chemotherapy in head and neck cancer (MACH-NC): An update on 107 randomized trials and 19,805 patients, on behalf of MACH-NC Group. Radiother Oncol. (2021) 156:281–93. doi: 10.1016/j.radonc.2021.01.013 PMC8386522 33515668

[2] Adelstein DJ Li Y Adams GL Wagner H Jr Kish JA Ensley JF. An intergroup phase III comparison of standard radiation therapy and two schedules of concurrent chemoradiotherapy in patients with unresectable squamous cell head and neck cancer. J Clin Oncol. (2003) 21:92–8. doi: 10.1200/JCO.2003.01.008 12506176

[3] Forastiere AA Goepfert H Maor M Pajak TF Weber R Morrison W. Concurrent chemotherapy and radiotherapy for organ preservation in advanced laryngeal cancer. N Engl J Med. (2003) 349:2091–8. doi: 10.1056/NEJMoa031317 14645636

[4] Porceddu SV Haddad RI. Management of elderly patients with locoregionally confined head and neck cancer. Lancet Oncol. (2017) 18:e274–83. doi: 10.1016/S1470-2045(17)30229-2 28456589

[5] Kunkler IH Audisio R Belkacemi Y Betz M Gore E Hoffe S. Review of current best practice and priorities for research in radiation oncology for elderly patients with cancer: the International Society of Geriatric Oncology (SIOG) task force. Ann Oncol. (2014) 25:2134–46. doi: 10.1093/annonc/mdu104 24625455

[6] Syrigos KN Karachalios D Karapanagiotou EM Nutting CM Manolopoulos L Harrington KJ. Head and neck cancer in the elderly: an overview on the treatment modalities. Cancer Treat Rev. (2009) 35:237–45. doi: 10.1016/j.ctrv.2008.11.002 19100689

[7] Sarris EG Harrington KJ Saif MW Syrigos KN. Multimodal treatment strategies for elderly patients with head and neck cancer. Cancer Treat Rev. (2014) 40:465–75. doi: 10.1016/j.ctrv.2013.10.007 24238923

[8] Maggiore R Zumsteg ZS BrintzenhofeSzoc K Trevino KM Gajra A Korc-Grodzicki B. The older adult with locoregionally advanced head and neck squamous cell carcinoma: knowledge gaps and future direction in assessment and treatment. Int J Radiat Oncol Biol Phys. (2017) 98:868–83. doi: 10.1016/j.ijrobp.2017.02.022 PMC5769962 28602414

[9] Ishii R Ohkoshi A Kiyota N Matsuura K Yasuda K Imamura Y. Management of elderly patients with head and neck cancer. Jpn J Clin Oncol. (2022) 52:313–21. doi: 10.1093/jjco/hyac013 35165732

[10] Oosting SF Desideri I Staelens D Caballero C Tribius S Simon C. Treatment patterns in older patients with locally advanced head and neck squamous cell carcinoma: Results from an EORTC led survey. J Geriatr Oncol. (2021) 12:1261–5. doi: 10.1016/j.jgo.2021.05.007 33994150

[11] Ahn MJ D’Cruz A Vermorken JB Chen JP Chitapanarux I Dang HQ. Clinical recommendations for defining platinum unsuitable head and neck cancer patient populations on chemoradiotherapy: A literature review. Oral Oncol. (2016) 53:10–6. doi: 10.1016/j.oraloncology.2015.11.019 26712252

[12] de Castro G Jr Alves GV Castro AF Chaves ALF De Marchi P de Oliveira TB. Criteria for eligibility to cisplatin in the curative treatment of head and neck cancer: Consensus opinion from a panel of experts. Crit Rev Oncol Hematol. (2018) 131:30–4. doi: 10.1016/j.critrevonc.2018.08.009 30293703

[13] Abdulla M Belal AA Sakr A El Arab LE Mokhtar M Allahloubi N. Eligibility criteria to cisplatin in head and neck squamous cell carcinoma: Egyptian expert opinion. Health Sci Rep. (2023) 6:e1037. doi: 10.1002/hsr2.1037 36698712 PMC9847398

[14] United Nations. World Population Ageing 2019 Highlights (2019). Available online at: https://www.un-ilibrary.org/content/books/9789210045537 (Accessed Jan 2024).

[15] World Health Organization. World health statistics 2022: monitoring health for the SDGs, sustainable development goals. Available online at: https://www.who.int/publications/i/item/9789240051157 (Accessed May 2024).

[16] Japan Society for Head and Neck Cancer. Report of Head and Neck Cancer Registry of Japan clinical statistics of registered patients, 2019 (2022). Available online at: http://www.jshnc.umin.ne.jp/report.html (Accessed Jan 2024).

[17] Charlson ME Pompei P Ales KL MacKenzie CR. A new method of classifying prognostic comorbidity in longitudinal studies: development and validation. J Chronic Dis. (1987) 40:373–83. doi: 10.1016/0021-9681(87)90171-8 3558716

[18] Yasuda K Uchinami Y Kano S Taguchi J Kawakita D Kitayama M. Radiotherapy with or without chemotherapy for locally advanced head and neck cancer in elderly patients: analysis of the Head and Neck Cancer Registry of Japan. Int J Clin Oncol. (2024) 29:241–7. doi: 10.1007/s10147-023-02450-7 38155239

[19] Ouchi Y Rakugi H Arai H Akishita M Ito H Toba K. Redefining the elderly as aged 75 years and older: Proposal from the Joint Committee of Japan Gerontological Society and the Japan Geriatrics Society. Geriatr Gerontol Int. (2017) 17:1045–7. doi: 10.1111/ggi.2017.17.issue-7 28670849

[20] Kiyota N Tahara M Mizusawa J Kodaira T Fujii H Yamazaki T. Weekly cisplatin plus radiation for postoperative head and neck cancer (JCOG1008): A multicenter, noninferiority, phase II/III randomized controlled trial. J Clin Oncol. (2022) 40:1980–90. doi: 10.1200/JCO.21.01293 PMC9197353 35230884

[21] Sharma A Kumar M Bhasker S Thakar A Pramanik R Biswas A. An open-label, noninferiority phase III RCT of weekly versus three weekly cisplatin and radical radiotherapy in locally advanced head and neck squamous cell carcinoma (ConCERT trial). J Clin Oncol. (2022) 40:6004–4. doi: 10.1200/JCO.2022.40.16_suppl.6004

[22] UChinami Y Yasuda K Kano S Otsuka M Hamada S Suzuki T. Treatment outcomes of radiotherapy with concurrent weekly cisplatin in older patients with locally advanced head and neck squamous cell carcinoma. Discovery Oncol. (2023) 14:226. doi: 10.1007/s12672-023-00844-7 PMC10709267 38063923

[23] Ghosh-Laskar S Kalyani N Gupta T Budrukkar A Murthy V Sengar M. Conventional radiotherapy versus concurrent chemoradiotherapy versus accelerated radiotherapy in locoregionally advanced carcinoma of head and neck: Results of a prospective randomized trial. Head Neck. (2016) 38:202–7. doi: 10.1002/hed.23865 25224814

[24] Dale W Williams GR MacKenzie AR Soto-Perez-de-Celis E Maggiore RJ Merrill JK. How is geriatric assessment used in clinical practice for older adults with cancer? A survey of cancer providers by the American Society of Clinical Oncology. JCO Oncol Pract. (2021) 17:336–44. doi: 10.1200/OP.20.00442 PMC8462667 33064058

[25] Hamaker M Lund C Te Molder M Soubeyran P Wildiers H van Huis L. Geriatric assessment in the management of older patients with cancer - A systematic review (update). J Geriatr Oncol. (2022) 13:761–77. doi: 10.1016/j.jgo.2022.04.008 35545495

[26] Anwar MR Yeretzian ST Ayala AP Matosyan E Breunis H Bote K. Effectiveness of geriatric assessment and management in older cancer patients: a systematic review and meta-analysis. J Natl Cancer Inst. (2023) 115:1483–96. doi: 10.1093/jnci/djad200 37738290

[27] Patil VM Noronha V Menon N Singh A Ghosh-Laskar S Budrukkar A. Results of phase III randomized trial for use of docetaxel as a radiosensitizer in patients with head and neck cancer, unsuitable for cisplatin-based chemoradiation. J Clin Oncol. (2023) 41:2350–61. doi: 10.1200/JCO.22.00980 36706347

[28] Arai M Ohno I Takahashi K Fan MM Tawada A Ishioka C. Current status of medical oncology in Japan and changes over the most recent 7-year period: results of a questionnaire sent to designated cancer care hospitals. Jpn J Clin Oncol. (2021) 51:1622–27. doi: 10.1093/jjco/hyab135 PMC8558914 34414432

