# Corrigendum: Indications for chemoradiotherapy in older patients with locally advanced head and neck cancer in Japan: a questionnaire survey in the JCOG head and neck cancer study group

**DOI:** 10.3389/fonc.2025.1558600

**Published:** 2025-04-10

**Authors:** Koichi Yasuda, Naomi Kiyota, Kazuto Matsuura, Satoshi Saito, Yoshitaka Honma, Yoshinori Imamura, Kaoru Tanaka, Sadamoto Zenda, Takuma Onoe, Takeshi Kodaira, Satoshi Kobayashi, Hidefumi Aoyama, Nobuhiro Hanai, Akihiro Homma

**Affiliations:** ^1^ Department of Radiation Oncology, Hokkaido University Hospital, Sapporo, Japan; ^2^ Department of Medical Oncology and Hematology, Cancer Center, Kobe University Hospital, Kobe, Japan; ^3^ Department of Head and Neck Surgery, National Cancer Center Hospital East, Kashiwa, Japan; ^4^ Department of Radiation Oncology, Saitama Medical University International Medical Center, Hidaka, Japan; ^5^ Department of Head and Neck, Esophageal Medical Oncology, National Cancer Center Hospital, Tokyo, Japan; ^6^ Department of Medical Oncology and Hematology, Kobe University Hospital, Kobe, Japan; ^7^ Department of Medical Oncology, Kindai University Faculty of Medicine, OsakaSayama, Japan; ^8^ Department of Radiation Oncology and Particle Therapy, National Cancer Center Hospital East, Kashiwa, Japan; ^9^ Division of Medical Oncology, Hyogo Cancer Center, Akashi, Japan; ^10^ Department of Radiation Oncology, Aichi Cancer Center Hospital, Aichi, Japan; ^11^ Department of Gastroenterology, Kanagawa Cancer Center, Yokohama, Japan; ^12^ Department of Radiation Oncology, Faculty of Medicine and Graduate School of Medicine, Hokkaido University, Sapporo, Japan; ^13^ Department of Head and Neck Surgery, Aichi Cancer Center Hospital, Nagoya, Japan; ^14^ Department of Otolaryngology-Head & Neck Surgery, Faculty of Medicine and Graduate School of Medicine, Hokkaido University, Sapporo, Japan

**Keywords:** older patients, head and neck cancer, chemoradiotherapy, radiotherapy, cisplatin

In the published article, **Supplementary Materials 1** and **2** were mistakenly not included in the publication. The missing material appears below.

The authors apologize for this error and state that this does not change the scientific conclusions of the article in any way. The original article has been updated.

