# Supplementary figures and images for: Indications for chemoradiotherapy in older patients with locally advanced head and neck cancer in Japan: a questionnaire survey in the JCOG head and neck cancer study group
Source: Front Oncol. 2025 Jan 8;14:1441056. doi: 10.3389/fonc.2024.1441056 (PMC11750991; doi:10.3389/fonc.2024.1441056)

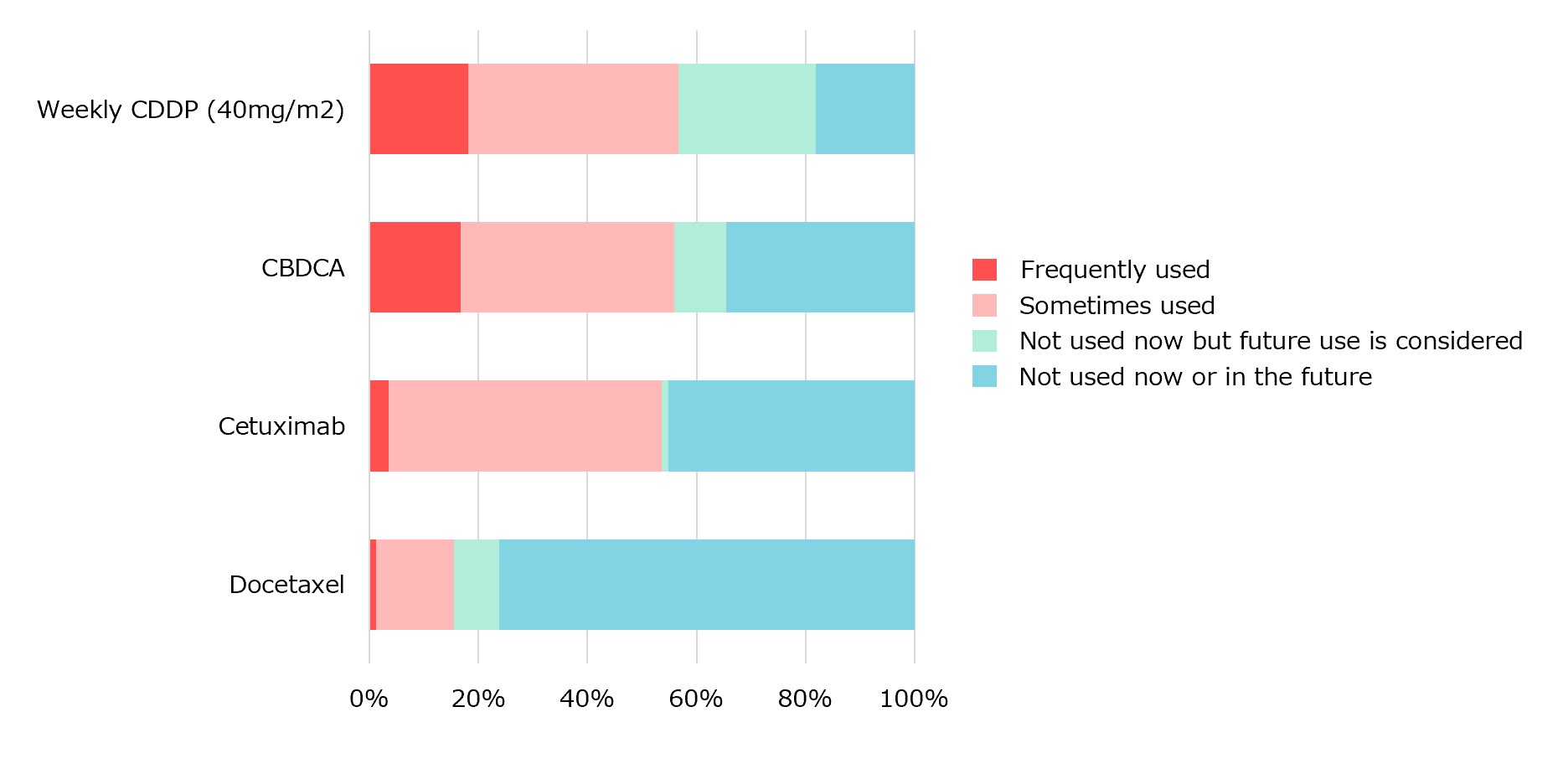

Supplement: Supplementary file 1 [file Image1.jpeg]

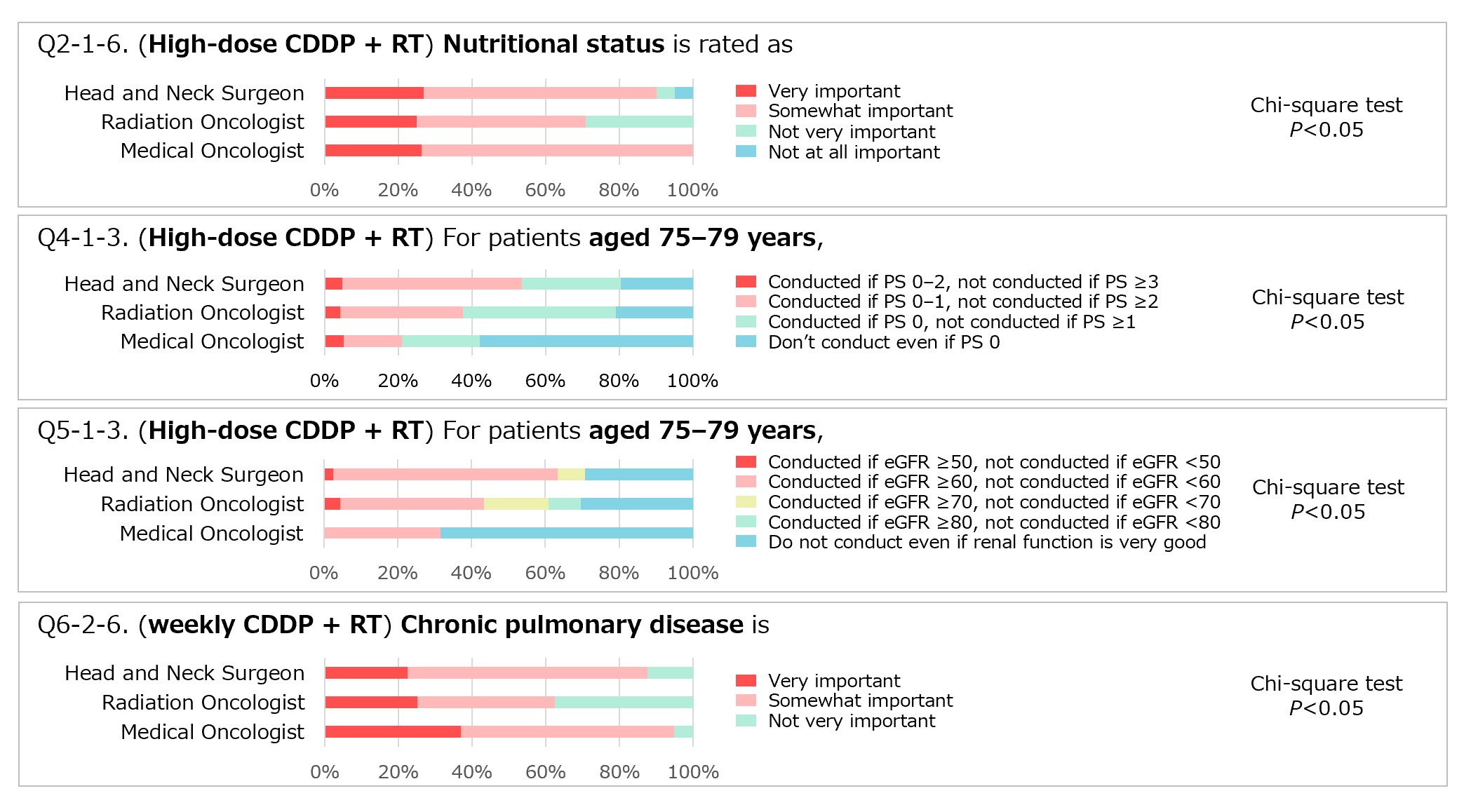

Supplement: Supplementary file 2 [file Image2.jpeg]
