# Supplementary material for: Indications for chemoradiotherapy in older patients with locally advanced head and neck cancer in Japan: a questionnaire survey in the JCOG head and neck cancer study group
Source: Front Oncol. 2025 Jan 8;14:1441056. doi: 10.3389/fonc.2024.1441056 (PMC11750991; doi:10.3389/fonc.2024.1441056)
Supplement: Supplementary file 3 [file SupplementaryFile1.pdf]

A list of participating institutions

Hokkaido University Hospital
Iwate Medical University
Tohoku University Hospital
Miyagi Cancer Center
Jichi Medical University
Saitama Medical University International Medical Center
National Cancer Center Hospital East
National Cancer Center Hospital
Tokyo Medical University Hospital
Tokyo Medical Center
Keio University Hospital
Tokyo Medical and Dental University
The Jikei University Hospital
Cancer Institute Hospital
The University of Tokyo School of Medicine
Yokohama City University Hospital
Tokai University School of Medicine
Gifu University School of Medicine
Shizuoka Cancer Center
Aichi Cancer Center
Nagoya University School of Medicine
Fujita Health University
Aichi Medical University Hospital
Kyoto University Hospital
Kyoto Prefectural University of Medicine
Kindai University Hospital
Osaka International Cancer Institute
Kansai Medical University Hospital
Kobe University School of Medicine
Hyogo Cancer Canter
Nara Medical University
Okayama University Hospital
Hiroshima University Hospital
National Hospital Organization Shikoku Cancer Center
National Hospital Organization Kyushu Cancer Center
Kyushu University Hospital
