# Supplementary material for: Indications for chemoradiotherapy in older patients with locally advanced head and neck cancer in Japan: a questionnaire survey in the JCOG head and neck cancer study group
Source: Front Oncol. 2025 Jan 8;14:1441056. doi: 10.3389/fonc.2024.1441056 (PMC11750991; doi:10.3389/fonc.2024.1441056)
Supplement: Supplementary file 4 [file SupplementaryFile2.pdf]

Questionnaire on chemoradiation therapy for head and neck cancer in older people

(The survey was conducted in Japan. This is the English translation.)

Q1. Do you have an upper age limit while considering whether to administer high-dose CDDP + RT (Q1-1), less-toxic chemotherapy + RT, or RT alone (Q1-2) in older people? If so, what are their ages?

Q1-1. High-dose CDDP + RT for older people

☐ No upper age limit is set (The treatment is given at any age as long as other conditions are favorable)
☐ An upper age limit is set. Usually, treatment is not administered to patients aged ___ or older.
☐ Other (free description: __________)

Q1-2. Less-toxic chemotherapy + RT or RT alone in older patients who are ineligible for high-dose CDDP + RT

☐ No upper age limit is set (The treatment is given at any age as long as other conditions are favorable)
☐ An upper age limit is set. Usually, treatment is not administered to patients aged ___ or older.
☐ Other (free description: __________)

Q2. How important are the following factors in deciding the indications for the treatment of older patients?

Q2-1. High-dose CDDP + RT for older patients

Q2-1-1. Performance Status (PS) is rated as [very important/somewhat important/not very important/not at all important].
Q2-1-2. Renal function is rated as [very important/somewhat important/not very important/not at all important].
Q2-1-3. Comorbidity is rated as [very important/somewhat important/not very important/not at all important].
Q2-1-4. Cognitive function is rated as [very important/somewhat important/not very important/not at all important].
Q2-1-5. Activities of daily living are rated as [very important/somewhat important/not very important/not at all important].
Q2-1-6. Nutritional status is rated as [very important/somewhat important/not very important/not at all important].

Q2-1-7. Polypharmacy is rated as [very important/somewhat important/not very important/not at all important].
Q2-1-8. Emotional (depressive) status is rated as [very important/somewhat important/not very important/not at all important].
Q2-1-9. Social (family) support is rated as [very important/somewhat important/not very important/not at all important].
Q2-1-10. Geriatric assessment tools are rated as [very important/somewhat important/not very important/not at all important].
Q2-1-11. Other (free description: __________)

Q2-2. Less-toxic chemotherapy + RT for older patients who are not eligible for high-dose CDDP + RT
Q2-2-1. Performance Status (PS) is rated as [very important/somewhat important/not very important/not at all important].
Q2-2-2. Renal function is rated as [very important/somewhat important/not very important/not at all important].
Q2-2-3. Comorbidity is rated as [very important/somewhat important/not very important/not at all important].
Q2-2-4. Cognitive function is rated as [very important/somewhat important/not very important/not at all important].
Q2-2-2. Activities of daily living are rated as [very important/somewhat important/not very important/not at all important].
Q2-2-6. Nutritional status is rated as [very important/somewhat important/not very important/not at all important].
Q2-2-7. Polypharmacy is rated as [very important/somewhat important/not very important/not at all important].
Q2-2-8. Emotional (depressive) status is rated as [very important/somewhat important/not very important/not at all important].
Q2-2-9. Social (family) support is rated as [very important/somewhat important/not very important/not at all important].
Q2-2-10. Geriatric assessment tools are rated as [very important/somewhat important/not very important/not at all important].
Q2-2-11. Other (free description: __________)

Q3. If high-dose CDDP + RT is not feasible, how often is less-toxic chemotherapy (Weekly-CDDP, cetuximab, CBDCA, and docetaxel) + RT administered to older patients?

Q3-1. Weekly CDDP + RT was classified as [frequently used/sometimes used/not used now but future use is considered/not used now or in the future].

Q3-2. Cetuximab was classified as [frequently used/sometimes used/not used now but future use is considered/not used now or in the future].

Q3-3. CBDCA was classified as [frequently used/sometimes used/not used now but future use is considered/not used now or in the future].

Q3-4. Docetaxel was classified as [frequently used/sometimes used/not used now but future use is considered/not used now or in the future].

Q4. How does PS affect your decisions regarding the indications for the treatment of older patients? (assuming that all conditions other than PS were favorable).

Q4-1. High-dose CDDP + RT for older patients

Q4-1-1. For patients aged 65–69 years, [do not conduct even if PS 0/conducted if PS 0, not conducted if PS ≥1/conducted with PS 0–1, not conducted if PS ≥2/conducted if PS 0–2, not conducted if PS ≥3].

Q4-1-2. For patients aged 70–74 years, [do not conduct even if PS 0/conducted if PS 0, not conducted if PS ≥1/conducted with PS 0–1, not conducted if PS ≥2/conducted if PS 0–2, not conducted if PS ≥3].

Q4-1-3. For patients aged 75–79 years, [do not conduct even if PS 0/conducted if PS 0, not conducted if PS ≥1/conducted with PS 0–1, not conducted if PS ≥2/conducted if PS 0–2, not conducted if PS ≥3].

Q4-1-4. For patients aged ≥80 years, [do not conduct even if PS 0/conducted if PS 0, not conducted if PS ≥1/conducted with PS 0–1, not conducted if PS ≥2/conducted if PS 0–2, not conducted if PS ≥3].

Q4-2. Less toxic chemotherapy + RT for older patients who are not eligible for high-dose CDDP + RT

Q4-2-1. For patients aged 65–69 years, [do not conduct even if PS 0/conducted if PS 0, not conducted if PS ≥1/conducted with PS 0–1, not conducted if PS ≥2/conducted if PS 0–2, not conducted if PS ≥3].

Q4-2-2. For patients aged 70–74 years, [do not conduct even if PS 0/conducted if PS 0, not conducted if PS ≥1/conducted with PS 0–1, not conducted if PS ≥2/conducted if PS 0–2, not conducted if PS ≥3].

Q4-2-3. For patients aged 75–79 years, [do not conduct even if PS 0/conducted if PS 0, not conducted if PS ≥1/conducted with PS 0–1, not conducted if PS ≥2/conducted if PS 0–2, not conducted if PS ≥3].

Q4-2-4. For patients aged ≥80 years, [do not conduct even if PS 0/conducted if PS 0, not conducted if PS ≥1/conducted with PS 0–1, not conducted if PS ≥2/conducted if PS 0–2, not conducted if PS ≥3].

Q5. How does renal function affect decisions regarding the indications for treatment in older patients? (assuming that all conditions other than renal function were favorable)

Q5-1. High-dose CDDP + RT for older patients

Q5-1-1. For patients aged 65–69 years, [do not conduct even if renal function is very good/conducted if eGFR ≥80, not conducted if eGFR <80/conducted if eGFR ≥70, not conducted if eGFR <70/conducted if eGFR ≥60, not conducted if eGFR <60/conducted if eGFR ≥50, not conducted if eGFR <50/conducted if eGFR ≥40, not conducted if eGFR <40].

Q5-1-2. For patients aged 70–74 years, [do not conduct even if renal function is very good/conducted if eGFR ≥80, not conducted if eGFR <80/conducted if eGFR ≥70, not conducted if eGFR <70/conducted if eGFR ≥60, not conducted if eGFR <60/conducted if eGFR ≥50, not conducted if eGFR <50/conducted if eGFR ≥40, not conducted if eGFR <40].

Q5-1-3. For patients aged 75–79 years, [do not conduct even if renal function is very good/conducted if eGFR ≥80, not conducted if eGFR <80/conducted if eGFR ≥70, not conducted if eGFR <70/conducted if eGFR ≥60, not conducted if eGFR <60/conducted if eGFR ≥50, not conducted if eGFR <50/conducted if eGFR ≥40, not conducted if eGFR <40].

Q5-1-4. For patients aged ≥80 years, [do not conduct even if renal function is very good/conducted if eGFR ≥80, not conducted if eGFR <80/conducted if eGFR ≥70, not conducted if eGFR <70/conducted if eGFR ≥60, not conducted if eGFR <60/conducted if eGFR ≥50, not conducted if eGFR <50/conducted if eGFR ≥40, not conducted if eGFR <40].

Q5-2. Weekly CDDP + RT for older patients who are not eligible for high-dose CDDP + RT

Q5-2-1. For patients aged 65–69 years, [do not conduct even if renal function is very good/conducted if eGFR ≥80, not conducted if eGFR <80/conducted if eGFR ≥70, not conducted if eGFR <70/conducted if eGFR ≥60, not conducted if eGFR <60/conducted if eGFR ≥50, not conducted if eGFR <50/conducted if eGFR ≥40, not conducted if eGFR <40].

Q5-2-2. For patients aged 70–74 years, [do not conduct even if renal function is very good/conducted if eGFR ≥80, not conducted if eGFR <80/conducted if eGFR ≥70, not conducted if eGFR <70/conducted if eGFR ≥60, not

conducted if eGFR <60/conducted if eGFR ≥50, not conducted if eGFR <50/conducted if eGFR ≥40, not conducted if eGFR <40].

Q5-2-3. For patients aged 75–79 years, [do not conduct even if renal function is very good/conducted if eGFR ≥80, not conducted if eGFR <80/conducted if eGFR ≥70, not conducted if eGFR <70/conducted if eGFR ≥60, not conducted if eGFR <60/conducted if eGFR ≥50, not conducted if eGFR <50/conducted if eGFR ≥40, not conducted if eGFR <40].

Q5-2-4. For patients aged ≥80 years, [do not conduct even if renal function is very good/conducted if eGFR ≥80, not conducted if eGFR <80/conducted if eGFR ≥70, not conducted if eGFR <70/conducted if eGFR ≥60, not conducted if eGFR <60/conducted if eGFR ≥50, not conducted if eGFR <50/conducted if eGFR ≥40, not conducted if eGFR <40].

Q6. How important are the following comorbidities in deciding indications for treatment in older patients? (assuming that all conditions other than comorbidities were favorable)

-Very important: Even one comorbidity classified in this category discourages treatment.

- Somewhat important: One comorbidity classified in this category does not discourage treatment, but multiple comorbidities do.

-Not very important: The comorbidities classified in this category did not significantly affect the decision to undergo treatment.

Q6-1. High-dose CDDP + RT for older patients

Q6-1-1. Myocardial infarction is [very important/somewhat important/not very important].

Q6-1-2. Congestive heart failure is [very important/somewhat important/not very important].

Q6-1-3. Peripheral vascular disease is [very important/somewhat important/not very important].

Q6-1-4. Cerebrovascular disease is [very important/somewhat important/not very important].

Q6-1-5. Dementia is [very important/somewhat important/not very important].

Q6-1-6. Chronic pulmonary disease is[very important/somewhat important/not very important].

Q6-1-7. Rheumatologic disease is [very important/somewhat important/not very important].

Q6-1-8. Peptic ulcer disease is [very important/somewhat important/not very important].
Q6-1-9. Mild liver disease is [very important/somewhat important/not very important].
Q6-1-10. Moderate or severe liver disease is [very important/somewhat important/not very important].
Q6-1-11. Diabetes without chronic complications is [very important/somewhat important/not very important].
Q6-1-12. Diabetes with chronic complications is [very important/somewhat important/not very important].
Q6-1-13. Hemiplegia or paraplegia is [very important/somewhat important/not very important].
Q6-1-14. AIDS/HIV is [very important/somewhat important/not very important].

Q6-2. Weekly CDDP + RT for older patients who are not eligible for high-dose CDDP + RT

Q6-2-1. Myocardial infarction is [very important/somewhat important/not very important].
Q6-2-2. Congestive heart failure is [very important/somewhat important/not very important].
Q6-2-3. Peripheral vascular disease is [very important/somewhat important/not very important].
Q6-2-4. Cerebrovascular disease is [very important/somewhat important/not very important].
Q6-2-5. Dementia is [very important/somewhat important/not very important].
Q6-2-6. Chronic pulmonary disease is [very important/somewhat important/not very important].
Q6-2-7. Rheumatologic disease is [very important/somewhat important/not very important].
Q6-2-8. Peptic ulcer disease is [very important/somewhat important/not very important].
Q6-2-9. Mild liver disease is [very important/somewhat important/not very important].
Q6-2-10. Moderate or severe liver disease is [very important/somewhat important/not very important].
Q6-2-11. Diabetes without chronic complications is [very important/somewhat important/not very important].
Q6-2-12. Diabetes with chronic complications is [very important/somewhat important/not very important].
Q6-2-13. Hemiplegia or paraplegia is [very important/somewhat important/not very important].

Q6-2-14. AIDS/HIV is [very important/somewhat important/not very important].
